# 

**Published:** 1866-12

**Authors:** 


### CONTENTS.

ORIGINAL COMMUNICATIONS.

Lady Hydrogen .......... 519
Extracting Teeth .......... 528
Compensation .......... 533
Valedictory Address .......... 536
Spontaneous Pulp-Death .......... 541
Gold Foil. .......... 542

PROCEEDINGS OF SOCIETIES.

The Connecticut Valley Dental Association .......... 544
Discussions of the Third Annual Meeting of Central States Association .......... 546

EDITORIAL.

Nitrous Oxyd as an Anæsthetic .......... 556
A Funny Resolution .......... 561
Close of the Volume .......... 563
The Twenty-First Volume .......... 564
Obituary .......... 564

